# Household Coping Strategies During the COVID-19 Pandemic in Chile

**DOI:** 10.3389/fsoc.2021.728095

**Published:** 2021-08-31

**Authors:** Julieta Palma, Consuelo Araos

**Affiliations:** ^1^ Department of Sociology, Universidad Alberto Hurtado, Santiago, Chile; ^2^ Department of Sociology, Pontificia Universidad Católica de Chile, Santiago, Chile

**Keywords:** Chile, COVID-19, family coping strategies, female-headed households, household composition, multigenerational households, older people, survey analysis

## Abstract

Chile was severely hit by the COVID-19 pandemic. The implementation of social distancing measures strongly affected the Chilean economy: the unemployment rate grew rapidly as well as the proportion of population temporarily excluded from the labour force. This article analyses the strategies deployed by Chilean households to cope with the impact of the pandemic at the intersection with household structure and its socio-economics characteristics. Secondary data analysis from the Encuesta Social COVID-19 (COVID-19 Social Survey), carried out by the Chilean Ministry for Social Development and Families, were used to analyse the income-generating and expenditure-minimising strategies adopted by households during the early months (March to July of 2020) of the pandemic. The results show that 60.3% of households experienced a drop in family income, 70.3% indicated that they had to use at least one income-generating strategy, and 76.6% at least one expenditure-minimising strategy during the early months of the pandemic. Indebtedness and decapitalisation characterised most of the coping strategies adopted by households. While living in multigenerational households does not protect family members from declining economic well-being, older people living in one- and two-generation households were found to be least affected economically during the crisis. They were also less likely to resort to these coping strategies, insofar as their income was mainly secured from pensions. Although female-headed households did not show a greater reduction in income than male-headed households, they were more likely to adopt income-generating strategies. This article draws attention to the possible effects of decapitalisation and indebtedness on the long-term economic well-being of households with different structures, and the resulting inequalities in their capacity to recover from the effects of the pandemic. The findings suggest that having a source of family income that is not dependent on labour market flows is crucial in times of crises.

## Introduction

In countries with limited welfare provision, such as Chile, families play a central role in protecting individuals from critical situations. Flows of support among extended family members take place on a daily basis, even if they maintain an independent residence. The implementation of social distancing measures to face the COVID-19 pandemic in Chile, which included localised lockdowns and mandatory mobility restrictions, applied earlier and more stringently for older people (Servicio Nacional del Adulto Mayor, 2020), strained inter-household support networks. Evidence from research elsewhere suggests that the disruption of family flows of support, in combination with the effects of the crisis on the labour market, can have a significant impact on vulnerable population groups, exacerbating the unequal impact of the crisis (Cantillon et al., 2021; Seck et al., 2021).

With a first confirmed case on 3 March 2020, Chile was severely hit by the COVID-19 pandemic, reaching the highest rate of infections per capita globally by mid-June 2020 (Fuentes and Sanders, 2020). The peak of the first wave was on 12 June 2020, with 351.9 confirmed cases and 10.6 confirmed deaths per million people (7 day rolling average) (Our World in Data, 2021). After declaring a “state of constitutional exception”, the Chilean government established an indefinite curfew at national level in March 2020, and lockdowns were organised at municipal and regional levels, implying long periods of confinement, restrictions on intra- and inter-municipality mobility, school and nursery closures and the suspension of non-essential economic activities. Older people were most severely affected, both by higher rates of COVID-19 infection and mortality, and by the stricter and less flexible confinement measures applied to those aged over 75 (Herrera et al., 2021).

All these measures had a significant economic impact on the Chilean population. Official figures from the Central Bank of Chile show a contraction of GDP in 2020 of 5.8% (Banco Central, 2020). According to the International Labour Organisation (ILO), in June−August 2020, the unemployment rate reached 12.9%, affecting more than one million people (Montt et al., 2020). An unprecedented increase occurred in the population temporarily excluded from the labour force, which reached 1.8 million people in the same period. Women were more affected by job losses than men: by July 2020, women’s labour force participation rate had fallen by 27 percentage points, compared to 19 points for men (Bravo et at., 2020).

COVID-19 measures, such as the closure of nurseries and schools, and the introduction of remote working in a large number of occupations, generated a significant increase in the unpaid care workload, especially for women. According to a longitudinal survey conducted in July 2020, both men and women increased the average number of hours spent in housework and childcare compared to the situation before the pandemic (Bravo et al., 2020). While the growth in housework and childcare was slightly greater for men, women were still devoting 21 more hours than men to domestic labour and care work; the increase for home schooling was mainly absorbed by women (0.7 compared to 0.4 hours for men).

Three main social protection measures were introduced during the early months of the pandemic (Montt et al., 2020). The first was the Employment Protection Law, enacted in April 2020, which gave workers access to income through unemployment insurance if employers suspended their contract due to COVID-19 restrictions. This measure was widely criticised because unemployment insurance in Chile is largely based on workers’ contributions. The second measure was the COVID-19 Bonus, which in April 2020 delivered a one-off payment of US $80 per person to the poorest 60% of households. The third was the creation in June 2020 of an Emergency Family Income (EFI), aimed at vulnerable households (targeted according to various criteria), providing a benefit ranging from US $80 to US $580 depending on the number of people in the household. The EFI was paid monthly from May 2020 (Ministerio de Desarrollo Social y de la Familia, 2021). Alongside these social protection measures, parental leave was extended in July 2020 through Parental Preventive Medical Leave, which was granted initially for 30 days and could be extended for a further 30 days (Montt et al., 2020). Also in July 2020, the Chilean Congress authorised the first of three withdrawals, each of 10%, from pension funds (with a minimum withdrawal of US $1,370), resulting in a significant proportion of people being left without, or with much depleted, pension funds.

In addition, it is important to note that the Chilean welfare care regime is not universal. Most monetary and non-monetary transfers focus on the most vulnerable groups of the population, guaranteeing them a minimum income and an “ethical” family income. Due to the strong targeting and low amounts provided by these programmes, their scope was very limited and left an important part of the population unprotected during the economic crisis induced by the pandemic.

In this context, two population groups were found to be particularly vulnerable to the impacts of the COVID-19 pandemic: older people, due to their higher morbidity and mortality from the virus, as well as the effects of lockdowns and specific restrictions on their mobility; and women, due to job loss and the increase in unpaid care workloads resulting from confinement and the closure of nurseries and schools.

The aim of this article is to analyse household coping strategies during the COVID-19 pandemic in Chile, and to determine how they were related to household structure and socio-economics characteristics. Secondary analysis of data from the Encuesta Social COVID-19, carried out by the Ministry for Social Development and Families in Chile, was used to examine the income-generating and expenditure-minimising strategies deployed by households during the early months (March to July) of the COVID-19 pandemic. To take account of the uneven impact of the pandemic on different population groups, the household typology adopted considers the generational composition of households, distinguishing between older people aged 65 and above, adults aged 18–64 and children under the age of 18, and between female and male household heads using self-reporting criteria.

### Review of the Literature on Household Coping Strategies

Previous research on the subject in Latin America has shown that some households are better equipped than others to cope with the negative effects of different kinds of shocks (González de la Rocha, 1994, 1995; Moser, 1997, 1998). This variation is not only a result of the socio-economic characteristics of the household, but also its ability to recognise external constraints and organise its resources accordingly. This overview of the relevant literature on family coping strategies in different countries considers the strategies adopted by families in times of economic and health crises with reference to household composition.

One of the main criticisms levelled against the concept of family or household strategies and their use in economic decision-making within households is the failure of analysts to question the notion that the family (or household) group has intentionality, interests and rationality, independent of the individuals that compose it (Crow, 1989; Saraceno, 1989; Schmink, 1984). Feminist literature has pointed out that the concept of family strategy takes little account of the conflicting interests of family members, which differs according to gender (Moch et al., 1987). The situation is further complicated in the case of multigenerational households, which are very common in Latin America where considerations regarding the unequal distribution of power between generations and family units within the household must also be taken into account (Palma, 2018).

### Household Composition and Intergenerational Solidarity

An important body of Latin American research has analysed the survival strategies deployed by poor urban households during the debt crisis and the subsequent process of structural adjustment in the 1980s. González de la Rocha (1994, González de la Rocha 1995) identified the responses of poor households to the macro processes of economic and institutional change: intensification of wage work by increasing the number of people working per household, particularly through the incorporation of women into the labour market; adjustments in consumption patterns, with reference to housework intensification and the reduction in the level of consumption in areas such as education, health and clothing; participation in networks of mutual assistance; and changes in the composition of households.

In this context, household extension – based primarily on multigenerational coresidence – has long been a widespread strategy adopted by families to cope with crises, enabling them to save on housing costs and bring able-bodied members into the household to contribute to salaried and domestic work (Oswald, 1991; González de la Rocha, 1994; Lomnitz, 1975; Moser, 1997; 1998; González de la Rocha, 2004; Hintze, 2004; Palma, 2021). As extended households become better equipped to cope with economic hardship than nuclear households, they have been found to be concentrated among poor families (González de la Rocha, 1994; Raczynski, 2006).

More recently, Latin American research has highlighted the importance of intergenerational solidarity in coping with the effects of economic deprivation, and with flows of financial and non-financial support both within and between households (Araos and Siles, 2021; Arriagada, 2014; Palma, 2018). In Chile, flows of exchanges have been observed between extended family members living nearby, such as the daily circulation of young children to distribute care among siblings; the flows of care between older parents and their adult children; help with cleaning and maintenance; and financial support for the schooling of grandchildren (Araos and Siles, 2021). Moreover, multigenerational households have been shown to be increasingly important for young women, since the childcare support provided by family members enables them to enter and remain in the labour market (Palma and Scott, 2020). The proportion of women aged 20–29 who are married, cohabiting or lone mothers living in extended households increased from 38% in 1990 to 54% in 2011 (Palma and Scott, 2020).

Research on the debt crisis and fiscal austerity in southern Europe since 2008 highlighted the “cushioning” role of the extended family on the impact of unemployment, low wages and the reduction of welfare for the economic well-being of individuals (Marí-Klose and Escapa, 2015). In this context, older generations have been shown to provide financial support and childcare assistance to the younger generations, due to the availability of pensions and time. Similar evidence has been found for Greece (Dagkouli-Kyriakoglou, 2018), Italy (Floridi, 2020), Portugal (Frade and Coelho, 2015; O’Connell and Brannen, 2021; Pedroso de Lima, 2016) and Spain (Moreno-Mínguez, 2017).

Studies analysing the domestic impact of the COVID-19 pandemic show that family coping strategies rely heavily on intergenerational solidarity. Comparing the cases of England and South Africa, Cantillon et al. (2021) show that families with young children in the first country were strongly affected by the disruption to flows of support from grandparents to grandchildren during the pandemic. This was the result of the restrictions on the mobility since both generations reside more frequently in separate households. In South Africa, it was common for grandparents to live in multigenerational households. Social distancing did not, therefore, interrupt the flows of support that they provide in caring for grandchildren; they may even have increased. In addition, state pensions in South Africa allowed older adults to contribute financially to households affected by the unemployment of their adult members.

Intergenerational coresidence has been found to facilitate flows of support among family members during the current crisis in many other countries. In the United States, for example, the coresidence of young adults with their older parents increased as a result of the pandemic owing to the sharing of resources as a means of reducing expenditure (Gilligan et al., 2020). In Spain, although no evidence of an increase in intergenerational coresidence has been found, probably because the arrangement is already relatively widespread, the willingness of older generations to provide financial and care assistance to younger generations did increase (Ayuso et al., 2020). In Chile, a panel study showed that, during the first months of the lockdown, 10% of older adults moved to live with other family members (Herrera et al., 2021). The proportion of older adults living with adult children increased from 43 to 51%, and those living with grandchildren from 25 to 32%. This increase in intergenerational coresidence could be seen both as a compensation strategy to offset the social isolation of older people and as a support strategy to mitigate the financial crisis and the care needs of younger generations.

### Female-Headed Households

Latin American research conducted during the debt crisis in the 1980s and the subsequent process of structural adjustment stressed the relationship between female-headed households and poverty (Buvinić and Gupta, 1997). However, another body of research questioned the idea that households headed by women were the “poorest of the poor”, showing that these households were not necessarily worse off than male-headed households when poverty is understood as a multidimensional phenomenon (Chant, 1997). This approach also identified the importance of recognising the heterogeneity of female-headed households, drawing attention to the different composition of these households. It distinguished, for example, between those formed by lone mothers and those where older women headed an extended household (Chant, 1997). Using a similar approach, a study of female-headed households in 14 Latin American countries has shown that female headship alone is not necessarily related to more difficult living conditions, unless it is combined with unstable conjugal unions or single-lone motherhood (Liu et al., 2017). After controlling for family status, the study found that female heads were less likely to live in poor households than men in similar circumstances.

An earlier study noted that the position that women occupy within the household matters in understanding how female-headed households cope with poverty (Moser, 1997). Older female heads may seek to include additional members to improve their livelihoods, while young lone mothers may seek shelter in the households of better-off relatives when they cannot afford independent living. For this reason, research using measures of household headship based on a self- or proxy-reporting criterion – common to most of the population censuses and household surveys in Latin America – has limitations when used to analyse family coping strategies among female-headed households. Firstly, it does not necessarily account for the real number of households where a woman is the main economic provider. Secondly, it does not consider hidden female headship (Chant, 1997), mainly involving lone mothers who live as subfamilies within an extended household (Palma, 2018). These factors need to be taken into account, because both women’s economic power and the place they occupy within the household influence family coping strategies.

Much of the previous research on the coping strategies of female-headed households focused on developing countries. In Indonesia, the livelihood strategies of female-headed households were based on the reduction of household food consumption, and indebtedness to neighbours or relatives providing childcare (Mujahiddin and Mahadirka, 2019). A study of female heads of household facing the shock caused by a cyclone in Bangladesh identified changes in cooking practices and food consumption, requests for loans from neighbours and acquaintances, and the generation of alternative income through micro-commerce (Khan, 2013). An analysis of the case of older female heads of household in South Africa showed that they combined the resources from their state pension with the development of small informal businesses, such as the sale of fruits and sweets (Sidloyi, 2016). These older women thus became the main agents for the maintenance of multigenerational households. Recent evidence for three European countries confirms that women – particularly lone mothers – are the “main managers of poverty”, assuming a key role in feeding their families in times of crisis (O’Connell and Brannen, 2021).

Research on coping and survival strategies carried out by female-headed households during the COVID-19 pandemic received much less attention than the impact on their household income. For example, a qualitative study conducted in the United States emphasised the ability of single mothers to generate active strategies to earn a living and care for their children, while managing the tension between production and reproduction (Hertz et al., 2020). To a large extent, this strategy relied on existing capacities to create and sustain support networks that operate outside the labour market, based on family generosity and reciprocity rather than on support from the state.

A broad consensus exists around the notion that the economic impact of measures of social distancing, confinement and large-scale closures of workplaces and schools during the COVID-19 pandemic affected women to a greater extent than men, especially in female-headed households (Bidegain et al., 2020; Kabeer et al., 2021). The reduction of income from paid work, and the intensification of domestic work associated with the care of children, disabled and older people exacerbated the pre-existing care crisis, making it difficult for women to remain in or return to the labour market (Seck et al., 2021). In Chile, single-parent households headed by women were found to be the category most affected by the employment crisis during the pandemic, with a 40% reduction in employed members, compared to 24% in two-parent households headed by men (Bergallo et al., 2021). Implementation of the direct cash transfer program (EFI), which prioritises women in the allocation of benefits − 62% of beneficiary households are headed by women − only partially offset the loss of income from employment (Bergallo et al., 2021).

## Data, Methods and Hypotheses

The data source for this article is the Encuesta Social COVID-19, a panel survey carried out by the Ministry of Social Development in Chile to identify the social consequences of the COVID-19 pandemic on the living conditions of families. The survey is based on a random sample of nationally representative households: the first wave took place in July 2020 (4,426 households) and the second in November 2020 (3,333 households). This article analyses data from the first wave collected at the peak of the first outbreak of the pandemic in Latin America.

The analysis is based on a sub-sample of 4,044 households headed by individuals aged 20 and older. The sub-sample excluded 329 cases for which no income information was available, 31 cases with no information about the level of education of the household head, and 9 cases of households composed of elderly people and children.

The analysis contained two dependent variables: the number of income-generating strategies and the number of expenditure-minimising strategies carried out by households in Chile during the first months of the COVID-19 pandemic, as summarised in Table 1.

**TABLE 1:** Dependent and independent variables.

Variables	Definitions
Number of income-generating strategies	Income-generating strategies are measured in a continuous variable that includes the number of actions that household members are taking to increase income during the COVID-19 pandemic (range 0–9). The following actions are included in the survey:
	1. Sell goods, such as a car, appliances, furniture, etc.
	2. Use household savings
	3. Request a loan or credit from a bank or other financial institution
	4. Borrow money from family, friends, neighbours or acquaintances
	5. Withdraw money from a credit card or business home or use a line of credit
	6. Lease or sell properties, land, rooms, work tools, etc.
	7. Perform additional activities that generate new income
	8. Request a salary or payment advance
	9. Other
	The number of income-generating strategies carried out for each household is calculated and the information is categorised into three dummy variables: no strategies, one or two strategies, three or more strategies
Number of expenditure-minimising strategies	Expenditure-minimising strategies are measured in a continuous variable that includes the number of actions that household members are taking to reduce expenses during the COVID-19 pandemic (range 0–11). The following actions are included in the survey:
	1. Reduce spending on education
	2. Reduce health expenses, include mental and dental health
	3. Reduce food expenses
	4. Stop paying bills for water, gas, electricity
	5. Stop paying bills for phone, internet, or other communication services
	6. Reduce expenses or stop paying for heating, paraffin, firewood
	7. Stop paying the rent
	8. Renegotiate or stop paying the mortgage
	9. Stop paying condominium fees
	10. Renegotiate or stop paying other debts
	11. Other
	The number of expenditure-minimising strategies carried out by each household is calculated, and this information is categorised into three dummy variables: no strategies, one or two strategies, three or more strategies
Decrease in household income	This dummy variable identifies whether or not the household suffered a decrease in monthly income as a result of the COVID-19 pandemic (including salaries, income from businesses or paid activities, pensions, bonuses, leases, money contributed by relatives, etc.).
Household type	The type of household is measured by identifying the presence of individuals of different generations in the household: children (under 18), adults (18–64) and older people (65+), including the following dummy variables: one-generation households, adults; one-generation households, older people; two-generation households, adults and children; two-generation households, adults and older people; three-generation households
Sex of the household head	This dummy variable identifies whether or not the household head is a woman
Household size	Household size is included as a continuous variable
Family status of the household’s head	This dummy variable identifies whether or not the household head coresides with his/her husband/wife or cohabiting partner. The Encuesta Social COVID-19 does not have information on the marital status of individuals in the survey
Education of the household head	Education of the household head is recoded into three dummy variables: incomplete high school education, including people who have not finished the obligatory curriculum of 12 years of schooling; complete high school education, including people who have completed only the compulsory curriculum; and some or complete higher education
Household income quintile	The income quintile consists of five dummy variables that correspond to an ascending order of per capita household income based on the full sample of households. Quintile I represents the poorest 20% of households and Quintile V represents the wealthiest 20%
Urban area	A dummy variable that measures whether the household resides in urban areas
Macrozona	Geographic macrozone of residence is included through five dummy variables: North macrozone; Central macrozone; South macrozone; Extreme South Macrozone and Metropolitan Region (capital of the country)
Number of employed individuals within the household	This variable measures the number of individuals within the household who are part-time or full-time employed, including the following dummy variables: zero; one or two individuals; three or more individuals
Lockdown status of the residence zone	This variable measures the number of months of uninterrupted lockdown in the area of residence, using the following dummy variables: 2 months or more, less than 2 months; without lockdown
State support	A dummy variable that measures whether the household received any state support from the central government or municipality

The analysis also includes two main predictors: household type (based on the number of generations coresiding) and sex of the household head. The dummy variable for the sex of household head identifies whether or not the household head is a woman. The Encuesta Social COVID-19 uses a measure of household headship based on a self- or proxy-reporting criterion, which has some limitations. Feminist research has highlighted, for example, the risk of under-estimating the real magnitude of female headship, by making those women who are the main income contributors to their household invisible. Despite this limitation, previous analyses carried out by the authors showed that this measure of household headship is strongly related to owner-occupancy and economic measures of household headship (Palma, 2018). It has therefore been applied in the present study. Univariate and bivariate descriptive and binary regression techniques were used to analyses the variables. Based on the review of the literature, the article aimed to test three main hypotheses in the Chilean context:

### Hypothesis 1

Because multigenerational (mostly three-generation) households are better equipped to cope with economic shocks, they might be expected to require fewer adaptive strategies to maintain their living standards during the pandemic.

### Hypothesis 2

Since the income of the older adults is less dependent on fluctuations in the labour market, households composed of older adults (one-, two- or three-generation households) might be expected to require fewer adaptive strategies to maintain their living standards during the pandemic.

### Hypothesis 3

As female-headed households have suffered more than other types of households during the crisis and are especially active in delivering survival strategies, they might be expected to require more adaptive strategies to maintain their living standards during the pandemic than male-headed households.

## Results

The finding reported here evaluate the role played by household structure and socio-economic characteristics in explaining income-generating and expenditure-minimising strategies during the early months of the COVID-19 pandemic.

### Impact of the COVID-19 Pandemic on Household Economic Well-Being

**FIGURE 1:**
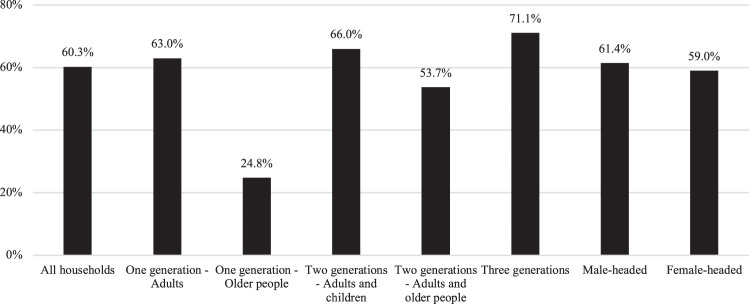
Households reporting a decrease in household income, by household composition and headship. Notes: Sample weights applied. Differences by household type are significant at *p* < 0.001. Differences by headship are not significant.

The descriptive analyses showed that the economic crisis generated by the pandemic had a significant impact on the economic well-being of households in Chile. As illustrated in Figure 1, 60.3% of households in the survey indicated that family income had decreased as a result of the pandemic. This decrease was most marked in one-generation households composed of adults, two-generation households composed of adults and children, and three-generation households. Households composed only of older people were the least economically affected as a result of the pandemic, followed by two-generation households composed of adults and older people. This observation does not mean that households composed of older people necessarily have a higher level of economic well-being than other types of households. Rather, it suggests that their income was more stable when the labour market was contracting, because it was derived mainly from pensions.

When the decrease in household income is analysed by household headship, no significant differences are observed according to whether the household is male headed or female headed. This finding contrasts with the available evidence showing that women were most likely to be affected by job loss during the crisis. One possible explanation for this finding is that female-headed compared to male-headed households implemented more – and more effective – income-generating strategies to compensate for decreasing household income.

### Household Strategies for Coping with the Economic Impact of the Pandemic

**FIGURE 2:**
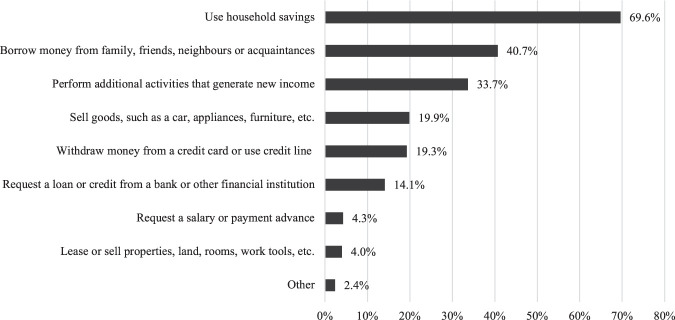
Types of income-generation strategies adopted by households as % of all households. Note: Sample weights applied.

In the context of decreasing household income, a significant proportion of households in Chile adopted various coping strategies, either to generate additional income or to reduce current expenditure. Of all households in Chile, 70.3% indicate that they had to use at least one income-generating strategy during the early months of the pandemic. As shown in Figure 2, the most common strategies were: the use of savings; receiving financial help from informal networks, such as relatives, friends and neighbours; carrying out additional activities to generate new income; and the sale of goods, such as a car, appliances or furniture. It is important to note that both the use of savings and the sale of household goods to generate additional income involve a process of decapitalisation of households. The generation of debt by withdrawing money using a credit card or credit line, bank loans or other forms of credit was not one of the most important income-generating strategies.

**FIGURE 3:**
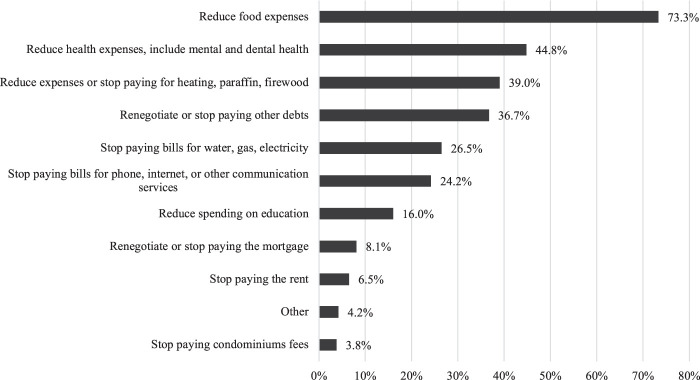
Types of expenditure minimising strategies carried out by households as % of all households. Note: Sample weights applied.

The analyses show that three-quarters of households in Chile adopted at least one expenditure-minimising strategy during the first months of the pandemic. As shown in Figure 3, these strategies were mainly aimed at reducing expenditure on basic goods and services, such as food, health, heating, water, gas and electricity, education and housing. In combination, ceasing to pay bills, mortgage/rent arrears and debts emerges as a widespread means of reducing expenses.

### Income-Generating and Expenditure-minimising Strategies by Household Composition and Headship

**FIGURE 4:**
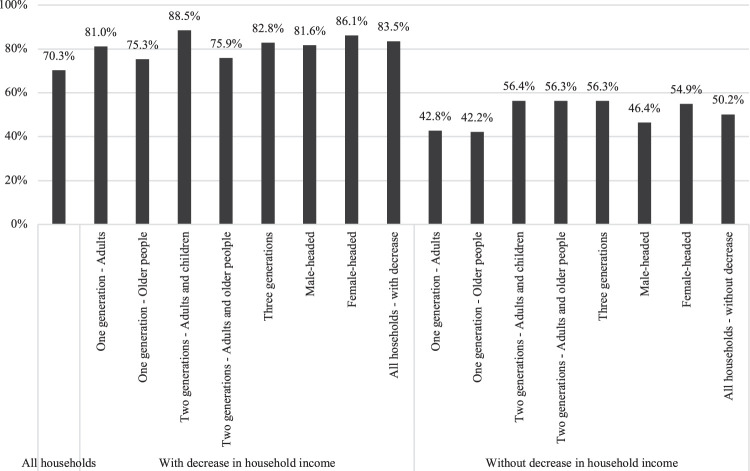
Households reporting having adopted at least one income-generating strategy by household composition and headship. Notes: Sample weights applied. Differences by income-generating strategies and household headship among households with decrease in household income are significant at *p* < 0.05. Differences by income-generating strategies and household headship among households without decrease in household income are significant at *p* < 0.01. Differences by income-generating strategies and household composition among households with decrease in household income are significant at *p* < 0.001. Differences by income-generating strategies and household composition among households without decrease in household income are significant at *p* < 0.001.

Since households were known to have suffered an unequal economic impact according to their composition and headship, it is important to examine whether the strategies adopted also differed according to these characteristics. Figure 4 shows the proportion of households that carried out at least one income-generating strategy according to these variables, differentiating between those that suffered a loss of income as a result of the COVID-19 pandemic and those that did not.

Households that experienced a decrease in their income were more likely to have carried out income-generating strategies than households that had not suffered a loss of income, irrespective of their composition or headship. Among the former, higher proportions were reported in one-generation households composed only of adults, two-generation households composed of adults and children and three-generational households. These results provide initial support for Hypothesis 2, which expected that households composed of older people would be less likely to adopt active strategies to increase income and reduce household expenses. When analysing the differences by household headship, households headed by women reported carrying out more income-generating strategies than households headed by men, whether or not they had suffered a decrease in their income, as anticipated in Hypothesis 3.

Importantly, a high proportion of households that had not experienced a decrease in their income also reported having implemented income-generating strategies during the pandemic. Given the cross-sectional nature of the data, it is not possible to know whether they were preventive-type strategies in a context of economic uncertainty, or whether these households did not suffer a loss of income because of the strategies implemented.

**FIGURE 5:**
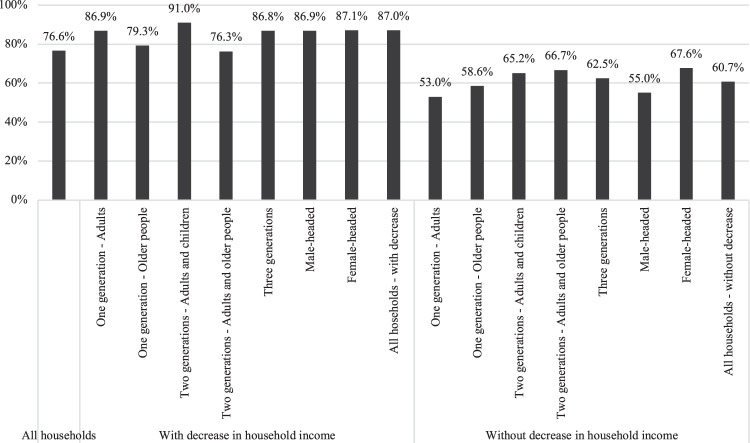
Households reporting having adopted at least one expenditure-minimising strategy by household composition and headship. Notes: Sample weights applied. All differences by income-generating strategies and household composition or household headship are significant at *p* < 0.001, except by differences by income-generating strategies and household headship among households with decrease in household income, which are significant at *p* < 0.01.

Figure 5 shows the proportion of households that have adopted at least one expenditure-minimising strategy according to their composition, headship and loss of income. As in the case of income-generating strategies, households that suffered a loss of income were more likely to adopt expenditure-minimising strategies than households that had not suffered such a loss, whatever its composition or headship. Most of households that experienced a loss of income carried out expenditure-minimising strategies, with only a slight decrease in the case of one-generation households composed of older people and two-generation households composed of adults and older people, as anticipated by Hypothesis 2. No significant differences were found by household headship. Among the households that did not suffer a loss of income, female-headed households, two-generation households composed of adults and older people, and those composed of adults with children registered the highest proportions.

### Factors Explaining Income-Generating and Expenditure-minimising Strategies Among Households

A binary logistic regression analysis was used to predict the performance of income-generation and expenditure-minimising strategies in a nationally representative sample of households (n = 4,044). Households that reported not having carried out these strategies during the first months of the pandemic were used as a reference category, in comparison to households having carried out at least one such strategy. An odds ratio of more than 1 means that the odds of an event occurring are higher for this category than in the reference category.

Table 2 displays the coefficients, the standard errors of B and the odds ratios of the binary logistic regression models carried out. One model was run for each type of household income-generating and expenditure-minimising strategies, including measures for household composition and headship, socio-economic and demographic controls.

**TABLE 2:** Binary logistic regressions predicting income-generating and expenditure-minimising strategies.

	Model 1			Model 2		
	Income-generating strategies			Expenditure-minimising strategies		
Predictors	Β	SE	OR	Β	SE	OR
**Household type** (Ref: One-generation households: Only adults)						
One-generation households: Only older people	**−0.423	0.164	0.655	*−0.403	0.174	0.668
Two-generation households: Adults and	0.138	0.120	1.148	0.151	0.129	1.163
children						
Two-generation households: Adults and older	*−0.260	0.131	0.771	**−0.471	0.139	0.625
people						
Three-generation households: Adults, children and	−0.372	0.200	0.690	*−0.524	0.218	0.592
older people						
**Household headship** (Ref: Male headship)						
Female headship	**0.270	0.090	1.309	0.165	0.095	1.179
**Household size**	*0.099	0.041	1.104	0.044	0.044	1.045
**Family status (household head)** (Ref: Without coresident husband/wife/cohabiting partner)						
With coresident husband/wife/cohabiting partner	0.011	0.100	1.011	−0.141	0.107	0.869
**Decrease in household income** (Ref: No decrease)	***1.281	0.085	3.599	***1.057	0.091	2.878
**Income quintile** (Ref: Quintile V)						
Quintile I (the poorest)	***1.404	0.174	4.071	***1.766	0.193	5.846
Quintile II	***1.509	0.158	4.523	***1.780	0.171	5.932
Quintile III	***1.290	0.148	3.632	***1.400	0.155	4.057
Quintile IV	***0.773	0.124	2.167	***0.812	0.125	2.251
**Number of employed individuals within the household** (Ref: None)						
One or two	−0.121	0.110	0.886	−0.141	0.122	0.868
Three or more	***−0.603	0.137	0.547	**−0.482	0.148	0.618
**Education (household head)** (Ref: Some or complete higher education)						
Incomplete high school education	**−0.338	0.124	0.713	0.035	0.132	1.035
Complete high school education	−0.116	0.108	0.891	0.112	0.112	1.119
**Lockdown status of the residence zone** (Ref: Without lockdown)						
Two months or more	0.247	0.182	1.280	*0.396	0.193	1.486
Less than two months	**0.289	0.111	1.334	0.137	0.119	1.147
**State support** (Ref: no support)	−0.094	0.089	0.910	**−0.248	0.096	0.781
**Urban zone** (Ref: rural)	−0.084	0.141	0.920	0.255	0.148	1.290
**Geographic macrozone** (Ref: Metropolitan Region)						
North macrozone	*0.375	0.164	1.455	*0.438	0.175	1.549
Central macrozone	0.203	0.183	1.225	*0.503	0.195	1.654
South macrozone	*0.431	0.183	1.538	0.359	0.192	1.432
Extreme South Macrozone	0.378	0.340	1.459	0.093	0.342	1.097
Intercept	***−1.020	0.260	0.360	**−0.831	0.276	0.436
Sample size			4.044			4.044
Cox & Snell R Square			0.191			0.168
Nagelkerke R Square			0.271			0.254

*Notes:* Sample weights applied. B = coefficiens; SE = standard errors; OR = odds ratio. *p < *0.05*. **p < *0.01*. ***p < *0.001*.

When compared to one-generation households composed only of adults (without children or older adults), one-generation households composed only of older people and two-generation households composed of adults and older people were less likely to have carried out income-generation strategies, net of other factors. These results provide some support for Hypothesis 2, which expected households composed of older people to be less likely to adopt active strategies to increase income and reduce household expenses. Contrary to what was expected in Hypothesis 1, three-generation households were found to be as likely as one-generation households composed of adults to carry out income-generating strategies.

As suggested by other research on the subject, female-headed households were more often found to be carrying out income-generating strategies than male-headed households. The odds of performing income-generating strategies in female-headed households were 30% higher than those registered in male-headed households. This finding supports Hypothesis 3, by showing that female-headed households, who were severely affected by the pandemic, were especially prone to adopt income-generating and expenditure-minimising strategies.

Household size had a significant positive effect on the odds of carrying out income-generating strategies. Larger households were more likely to carry out income-generating strategies, whereas the family status of the household head had no significant effect on the implementation of these strategies.

A strong association was observed between household income level and the odds of carrying out income-generating strategies. Households with higher income levels were more likely to perform this kind of strategy. The number of employed persons within the household had a negative effect on the implementation of income-generating strategies in households with three or more employed members, and they were less likely to carry out these strategies. Household heads with incomplete high school education were also less likely to carry out income-generating strategies.

The receipt of state financial support did not have a significant effect on the likelihood of carrying out income-generating strategies. The implementation of a lockdown lasting less than 2 months increased the odds of carrying out income-generating strategies.

Columns 5–7 of Table 2 show the results of the regression model for expenditure-minimising strategies. All household types, including those with older people, were less likely to carry out these strategies than one-generation households composed only of adults. For example, the odds of carrying out expenditure-minimising strategies in three-generation households were 40% lower than those for one-generation households composed only of adults. Two-generation households composed of adults and children did not show significant differences compared to the reference category. These results provide additional support for Hypothesis 2, which expected that households composed of older people would have less need for active strategies to reduce their household expenses. These results also confirm what was expected by Hypothesis 1, showing that three-generation households required fewer adaptive strategies to maintain their living standards during the pandemic.

Female headship did not have a strong significant effect (sig.= 0.083) on the odds of carrying out expenditure-minimising strategies once income level and other household characteristics have been taken into account. Interactions between female headship and household income quintile (not included in this article) also show no significant effect, except in the case of female-headed households of quintile IV, which are more likely to deploy expenditure-minimising strategies than male-headed households of the wealthiest (V) income quintile.

This finding would seem to contradict what is expected by Hypothesis 3 by suggesting that female-headed households are not particularly active in adopting strategies that seek to minimise expenditure, despite having been more severely affected by the crisis. This contradiction could be explained by the fact that these households were more often associated with the presence of children, which limited their options for reducing expenses. It is also possible to interpret this finding as a result of receiving child support from children’s fathers. However, it should be noted that family law in Chile does not guarantee that women and children receive economic support from their former husbands or fathers, as illustrated by the considerable number of claims for alimony that the courts receive each year, and the fact that the non-payment of alimony is the main cause of imprisonment in Chile (Palma, 2018). More research would be needed to test these additional hypotheses. No significant effect was found in relation to other household characteristics, such as household size or the family status of the household head.

As with income-generating strategies, a strong association was observed between household income level and expenditure-minimising strategies. Households with lower incomes were less likely to adopt these strategies than households with higher incomes. Having three or more employed members in the household reduced the odds, whereas the level of education of the head of the household did not have a significant effect. By contrast, receiving state financial support was found to have a significant negative effect on the likelihood of carrying out expenditure-minimising strategies. Although limited in scope and at a low level, households receiving state financial support were less likely to perform these types of strategies, whereas the implementation of localised lockdowns for two or more months increased the odds of carrying out expenditure-minimising strategies.

## Discussion and Conclusion

In the context of the Global South, Chile was one of the first countries to be affected by the retreat of the state from the provision of welfare. The reduction in social assistance and the privatisation of public services took effect during the dictatorship of Augusto Pinochet (1973–1990). The burden of welfare provision was transferred from the state to families and individuals, with dramatic consequences for the middle- and low-income groups. During the debt crisis and subsequent structural adjustment of the 1980s in Latin America, the lack of state support meant that almost half the population fell into poverty (Palma, 2018). The combined economic and public health crises of the 2020s placed even greater strain on the chances of survival for households.

The findings reported in this article provide new insights into the strategies adopted by households in Chile to cope with the economic effects of the COVID-19 pandemic. Previous literature on the subject, both in Latin America and in other countries, identified intra- and inter-household strategies involving support flows between extended family members. It highlighted the entry into the labour market of household members who were not employed prior to the crisis; the adjustments made in the consumption patterns of basic goods and services; reconfigurations in living arrangements, either to support vulnerable family members, or to incorporate members who contributed income or domestic work to the household, by increasing flows of financial support and the provision of care for grandchildren. The findings presented in this article confirm the value of these different strategies during the pandemic. They also identify other actions carried out by households, thereby contributing a more complete picture of the way in which households confront critical situations.

The use of household savings and the sale of household goods emerged as a widely used income-generating strategy among Chilean households. This finding suggests that a process of decapitalisation of households was taking place, resulting from the liquidation of assets to generate additional income to cope with the crisis. By depleting the assets accumulated by households during years of effort, the decapitalisation process raises questions about how households will survive over time.

A second significant finding concerns the generation of debt as a coping strategy, either to produce income or to reduce expenses. The results from the study show that indebtedness takes place both in the framework of informal networks − borrowing money from family, friends, neighbours or acquaintances − or, to a lesser extent, through the financial system. The analysis shows that a substantial proportion of households defaulted on servicing debts during the pandemic to reduce their expenditure.

Two aspects of the Chilean context contribute to an understanding of these findings. Firstly, the significant number of social services privatised during the Pinochet dictatorship (1973–1990), as a consequence of the neoliberal turn of the State, had increased the financial burden on households and their vulnerability to the present crisis. Secondly, the Chilean economy has been characterised in recent decades by a systematic process of household financialisation, largely affecting poor households (González López, 2018). This process has contributed to the diversification of forms of indebtedness, supplementing pre-existing informal structures of monetary flows.

Another finding from the research highlights the key role that older people played in developing household responses to the crisis. Except for the case of three-generation households, those composed of older people were found to be the least affected by the loss of economic well-being during the crisis. They were also less likely to resort to coping strategies, either to generate income or reduce expenditure. This finding is largely attributable to the fact that the income of older people was derived primarily from pensions, which protected them from labour market fluctuations. In view of the negative impact of some of the strategies identified in the article on the long-term economic well-being of households (decapitalisation and indebtedness), these households would seem to be better equipped to recover from the impact of the pandemic.

This finding does not mean that older people in Chile enjoy more favourable economic conditions than the rest of the population. On the contrary, the low level of pensions resulting from a system based on individual capitalisation mean that they live in conditions of extreme economic precariousness. Data obtained from the Superintendent for Pensions (Superintendencia de Pensiones, 2021) show that 50% of retired people in Chile received a pension below US $205 in 2020, an amount that represents half the legal minimum wage for that year (US $410). Although low, pensions provide a stable income stream for their households, which prevented their level of economic well-being from deteriorating further during the pandemic.

The findings from the study suggest that having a source of family income not dependent on labour market flows is crucial in times of crisis. Furthermore, this observation lends support to the literature on intergenerational relationships that highlights the role of older people in contributing to the well-being of younger generations, whether through financial support, assistance with childcare or housing. Despite being more exposed to the risk of contracting and dying from COVID-19, older people were found to be less at risk economically, unless they had depleted their pension funds by drawing down their reserves as authorised by the government in July 2020. This finding could usefully be taken into account in discussions by the Chilean Congress about the future reform of the pension system and the introduction of a universal minimum income.

In line with research in other countries on the social impacts of the COVID-19 pandemic, the findings reported here show the significant impact that the crisis had on women as household heads. They were more likely to carry out income-generating strategies, with adverse consequences for decapitalisation and indebtedness, although they were not more likely to implement expenditure-minimising strategies. A possible explanation for these somewhat unexpected finding is that the options for reducing expenses on basic goods and services, such as food, health, heating, water, gas and electricity, education and housing, were limited for the many female-headed households composed of lone mothers with children. Another explanation could be that female-headed households were used to getting by on a very low income, and to being in debt, as suggested by research on poor families in wealthy societies (O’Connell and Brannen, 2021).

Public health measures implemented early in the pandemic − localised lockdowns – were found to increase the likelihood of households adopting coping strategies. Lockdowns lasting less than two months made households more likely to implement strategies to generate additional income. Similarly, localised lockdowns lasting more than two months increased the likelihood of households adopting expenditure-minimising strategies. These findings provide evidence on the negative effect of measures that restrict people’s mobility on the domestic economy of households. By contrast, targeted financial aid in the early months of the pandemic, no matter how limited, made households less likely to adopt coping strategies aimed at reducing their consumption of basic goods and services and to generate debt when paying bills. These findings suggest that measures restricting mobility need to be accompanied by more generous universal financial assistance to compensate for the loss of household income.

The question that remains following this analysis of household strategies during the early months of the pandemic concerns the longer-term effectiveness of these strategies. The data for this article were collected in July 2020 when the duration of the pandemic was uncertain. Future research on the subject using longitudinal survey analysis will allow us to identify whether the implementation of income-generating and expenditure-minimising strategies was able to cushion the impact of the crisis on the economic well-being of households in the longer term, and to identify in what type of households these strategies were most effective. Future research will also reveal the medium and long-term effects of the process of household decapitalisation and indebtedness on their coping strategies in helping or hindering the post-pandemic recovery process.

## Data Availability

Publicly available datasets were analysed in this study. This data can be found here: Observatorio Social–Ministerio de Desarrollo Social y Familia [Social Observatory–Ministry for Social Development and Families] [http://observatorio.ministeriodesarrollosocial.gob.cl/encuesta-social-covid19-primera-ronda].
